# Bis[1,3-bis­(1-benzyl-1*H*-benzimidazol-2-yl)-2-oxapropane]zinc(II) dipicrate dimethyl­formamide disolvate

**DOI:** 10.1107/S1600536809022296

**Published:** 2009-06-17

**Authors:** Huilu Wu, Ruirui Yun, Ke Li, Sun Tao, Kaitong Wang

**Affiliations:** a School of Chemical and Biological Engineering, Lanzhou Jiaotong University, Lanzhou 730070, People’s Republic of China

## Abstract

In the title compound, [Zn(C_30_H_26_N_4_O)_2_](C_6_H_2_N_3_O_7_)_2_·2C_3_H_7_NO, the Zn^II^ ion is coordinated in a distorted octa­hedral environment involving four equatorial N atoms and two O atoms in axial sites. The dihedral angles between the benzimidazole ring system and the phenyl rings in each of the benzyl­benzimidazole units are 78.56 (12), 81.68 (11), 75.76 (10) and 85.78 (9)°. In the crystal structure, there are weak but significant inter­molecular π–π stacking inter­actions with centroid–centroid distances of 3.685 (1) and 3.978 (1) Å.

## Related literature

For the nickel(II) ethanol 0.25-solvate analog of the title compound, see: Wu *et al.* (2009 ▶).
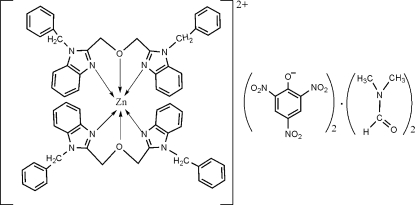


## Experimental

### 

#### Crystal data

[Zn(C_30_H_26_N_4_O)_2_](C_6_H_2_N_3_O_7_)_2_·2C_3_H_7_NO
*M* *_r_* = 1584.87
Monoclinic, 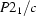

*a* = 13.3235 (2) Å
*b* = 18.1630 (3) Å
*c* = 30.0144 (5) Å
β = 97.267 (1)°
*V* = 7205.0 (2) Å^3^
*Z* = 4
Mo *K*α radiation
μ = 0.43 mm^−1^
*T* = 153 K
0.33 × 0.25 × 0.14 mm

#### Data collection

Rigaku R-AXIS SPIDER diffractometer
Absorption correction: multi-scan (*ABSCOR*; Higashi, 1995 ▶) *T*_min_ = 0.872, *T*_max_ = 0.943
65605 measured reflections
16426 independent reflections
11777 reflections with *I* > 2σ(*I*)
*R*_int_ = 0.039

#### Refinement

*R*[*F* ^2^ > 2σ(*F* ^2^)] = 0.041
*wR*(*F* ^2^) = 0.129
*S* = 1.15
16426 reflections
1019 parameters
H-atom parameters constrained
Δρ_max_ = 0.54 e Å^−3^
Δρ_min_ = −0.86 e Å^−3^

### 

Data collection: *RAPID-AUTO* (Rigaku/MSC, 2004 ▶); cell refinement: *RAPID-AUTO*; data reduction: *RAPID-AUTO* program(s) used to solve structure: *SHELXS97* (Sheldrick, 2008 ▶); program(s) used to refine structure: *SHELXL97* (Sheldrick, 2008 ▶); molecular graphics: *SHELXTL* (Sheldrick, 2008 ▶); software used to prepare material for publication: *SHELXTL* .

## Supplementary Material

Crystal structure: contains datablocks global, I. DOI: 10.1107/S1600536809022296/lh2840sup1.cif

Structure factors: contains datablocks I. DOI: 10.1107/S1600536809022296/lh2840Isup2.hkl

Additional supplementary materials:  crystallographic information; 3D view; checkCIF report

## Figures and Tables

**Fig. 1.**
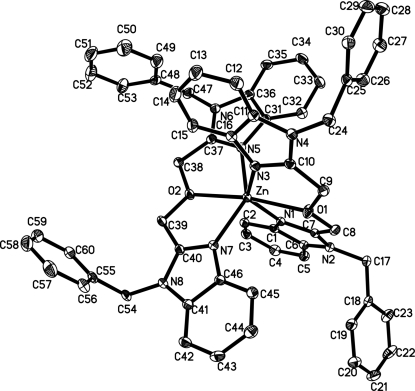
Molecular structure and atom numbering for the cation. Hydrogen atoms have been omitted for clarity and the displacement ellipsoids are shown at the 30% probability level.

**Fig. 2.**
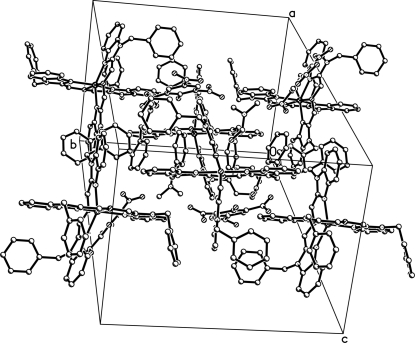
Part of the crystal structure. H atoms are not shown.

## supplementary crystallographic information

### Comment

For background information see Wu *et al.* (2009). The asymmetric unit of the title compound consists of a discrete di[1,3-bis(1-benzylbenzimidazol-2-yl)-2-oxapropane] zinc(II) cation (Fig.1), two picrate anions and two molecules of dimethylformamide. The Zn^II^ ion is six-coordinated with a N_4_O_2_ ligand set. The bobb (1,3-bis(1-benzylbenzimidazol-2-yl)-2-oxapropane) ligand acts as a tridentate donor. The coordination geometry of the Zn^II^ ion is distorted octahedral. This geometry is assumed by the Zn^II^ ion presumably to relieve steric crowding. In the crystal structure there are weak but significant intermolecular π–π stacking interactions with centroid to centroid distances of 3.685 (1) and 3.978 (1)Å (Fig. 2).

### Experimental

To a stirred solution of 1,3-bis(1-benzylbenzimidazol-2-yl)-2-oxapropane (0.183 g, 0.4 mmol) in hot MeOH (15 ml) was added Zn(C_6_H_2_N_3_O_7_)_2_ (0.104 g, 0.2 mmol) in MeOH (5 ml). A yellow crystalline product formed rapidly. The precipitate was filtered off, washed with MeOH and absolute Et_2_O, and dried *in vacuo*. The dried precipitate was dissolved in DMF resulting in a yellow solution. Yellow crystals suitable for X-ray diffraction studies were obtained by ether diffusion into a DMF solution of the title compound after three days at room temperature. Yield, 0.191 g (70%). (found: C, 59.20; H, 4.41; N,14.18. Calcd. for C78 H70 N16 Zn O18: C, 59.11; H, 4.45; N, 14.14)

### Refinement

All H atoms were found in difference Fourier maps and were subsequently refined in a riding-model approximation with fixed C—H distances ranging from 0.95 to 0.99Å and *U*_iso_(H) = 1.2 *U*_eq_ or *U*_iso_(H) = 1.5*U*_eq_ of the carrier atom, respectively.

### Crystal data

**Table:**

[Zn(C_30_H_26_N_4_O)_2_](C_6_H_2_N_3_O_7_)_2_·2C_3_H_7_NO	*F*(000) = 3296
*M**_r_* = 1584.87	*D*_x_ = 1.461 Mg m^−^^3^
Monoclinic, *P*2_1_/*c*	Mo *K*α radiation, λ = 0.71073 Å
Hall symbol: -P 2ybc	Cell parameters from 7749 reflections
*a* = 13.3235 (2) Å	θ = 3.2–27.5°
*b* = 18.1630 (3) Å	µ = 0.43 mm^−^^1^
*c* = 30.0144 (5) Å	*T* = 153 K
β = 97.267 (1)°	Block, yellow
*V* = 7205.0 (2) Å^3^	0.33 × 0.25 × 0.14 mm
*Z* = 4	

### Data collection

**Table:**

Rigaku R-AXIS SPIDER diffractometer	16426 independent reflections
Radiation source: fine-focus sealed tube	11777 reflections with *I* > 2σ(*I*)
graphite	*R*_int_ = 0.039
φ and ω scans	θ_max_ = 27.5°, θ_min_ = 3.0°
Absorption correction: multi-scan (*ABSCOR*; Higashi, 1995)	*h* = −17→17
*T*_min_ = 0.872, *T*_max_ = 0.943	*k* = −23→22
65605 measured reflections	*l* = −38→38

### Refinement

**Table:**

Refinement on *F*^2^	Secondary atom site location: difference Fourier map
Least-squares matrix: full	Hydrogen site location: inferred from neighbouring sites
*R*[*F*^2^ > 2σ(*F*^2^)] = 0.041	H-atom parameters constrained
*wR*(*F*^2^) = 0.129	*w* = 1/[σ^2^(*F*_o_^2^) + (0.067*P*)^2^ + 0.9168*P*] where *P* = (*F*_o_^2^ + 2*F*_c_^2^)/3
*S* = 1.15	(Δ/σ)_max_ = 0.018
16426 reflections	Δρ_max_ = 0.54 e Å^−^^3^
1019 parameters	Δρ_min_ = −0.86 e Å^−^^3^
0 restraints	Extinction correction: *SHELXL97* (Sheldrick, 2008), Fc^*^=kFc[1+0.001xFc^2^λ^3^/sin(2θ)]^-1/4^
Primary atom site location: structure-invariant direct methods	Extinction coefficient: 0.00109 (16)

### Special details

**Table:**

Geometry. All e.s.d.'s (except the e.s.d. in the dihedral angle between two l.s. planes) are estimated using the full covariance matrix. The cell e.s.d.'s are taken into account individually in the estimation of e.s.d.'s in distances, angles and torsion angles; correlations between e.s.d.'s in cell parameters are only used when they are defined by crystal symmetry. An approximate (isotropic) treatment of cell e.s.d.'s is used for estimating e.s.d.'s involving l.s. planes.
Refinement. Refinement of *F*^2^ against ALL reflections. The weighted *R*-factor *wR* and goodness of fit *S* are based on *F*^2^, conventional *R*-factors *R* are based on *F*, with *F* set to zero for negative *F*^2^. The threshold expression of *F*^2^ > σ(*F*^2^) is used only for calculating *R*-factors(gt) *etc*. and is not relevant to the choice of reflections for refinement. *R*-factors based on *F*^2^ are statistically about twice as large as those based on *F*, and *R*- factors based on ALL data will be even larger.

### Fractional atomic coordinates and isotropic or equivalent isotropic displacement parameters (Å^2^)

**Table:**

	*x*	*y*	*z*	*U*_iso_*/*U*_eq_	
Zn	0.638400 (17)	0.988839 (12)	0.247281 (7)	0.02001 (8)	
O1	0.71979 (11)	0.96447 (9)	0.32014 (5)	0.0313 (4)	
O2	0.53999 (13)	1.02713 (8)	0.18066 (5)	0.0314 (4)	
O3	0.69357 (13)	0.88365 (10)	−0.14192 (5)	0.0419 (4)	
O4	0.7828 (2)	1.01940 (12)	−0.14526 (6)	0.0719 (7)	
O5	0.78149 (16)	1.06606 (11)	−0.07982 (7)	0.0570 (5)	
O6	0.98063 (13)	0.92170 (10)	0.03757 (5)	0.0416 (4)	
O7	1.00246 (14)	0.80582 (10)	0.02480 (6)	0.0469 (5)	
O8	0.69600 (15)	0.70231 (10)	−0.07005 (6)	0.0505 (5)	
O9	0.71053 (13)	0.73280 (10)	−0.13856 (5)	0.0432 (4)	
O10	1.11818 (14)	0.57178 (10)	0.11553 (5)	0.0456 (5)	
O11	1.27610 (15)	0.62688 (15)	0.07752 (8)	0.0723 (7)	
O12	1.22390 (15)	0.72272 (12)	0.04024 (7)	0.0569 (5)	
O13	0.95381 (16)	0.66027 (10)	−0.08094 (6)	0.0515 (5)	
O14	0.83893 (15)	0.58166 (12)	−0.06673 (6)	0.0540 (5)	
O15	0.85306 (15)	0.46392 (11)	0.07245 (6)	0.0515 (5)	
O16	0.94946 (15)	0.50220 (11)	0.13070 (6)	0.0506 (5)	
O17	0.68321 (13)	0.07282 (10)	0.09630 (6)	0.0417 (4)	
O18	0.82449 (17)	0.73430 (15)	0.05674 (7)	0.0777 (7)	
N1	0.53073 (13)	0.97799 (9)	0.29215 (5)	0.0223 (4)	
N2	0.47479 (13)	0.96348 (9)	0.35851 (5)	0.0235 (4)	
N3	0.78731 (13)	0.95312 (9)	0.24456 (5)	0.0225 (4)	
N4	0.93027 (13)	0.89371 (9)	0.26902 (6)	0.0249 (4)	
N5	0.58236 (13)	0.89780 (9)	0.20769 (5)	0.0222 (4)	
N6	0.49619 (13)	0.83874 (9)	0.14987 (6)	0.0243 (4)	
N7	0.64458 (12)	1.10460 (9)	0.24216 (5)	0.0217 (4)	
N8	0.59794 (13)	1.21333 (9)	0.21164 (6)	0.0232 (4)	
N9	0.78936 (16)	1.01361 (12)	−0.10466 (7)	0.0394 (5)	
N10	0.96116 (14)	0.86512 (11)	0.01545 (6)	0.0327 (4)	
N11	0.72462 (14)	0.74446 (11)	−0.09777 (6)	0.0346 (4)	
N12	1.21010 (16)	0.66128 (14)	0.05444 (7)	0.0426 (5)	
N13	0.91676 (17)	0.61739 (12)	−0.05536 (6)	0.0393 (5)	
N14	0.92265 (15)	0.50290 (10)	0.09008 (7)	0.0340 (5)	
N15	0.83209 (15)	0.12038 (11)	0.07982 (6)	0.0330 (4)	
N16	0.86059 (16)	0.69339 (12)	0.12819 (7)	0.0429 (5)	
C1	0.42520 (15)	0.98170 (10)	0.28564 (7)	0.0212 (4)	
C2	0.35658 (15)	0.99316 (10)	0.24695 (7)	0.0230 (4)	
H2A	0.3792	0.9977	0.2183	0.028*	
C3	0.25569 (17)	0.99758 (11)	0.25170 (7)	0.0289 (5)	
H3A	0.2079	1.0053	0.2258	0.035*	
C4	0.22116 (17)	0.99103 (12)	0.29380 (8)	0.0308 (5)	
H4A	0.1508	0.9953	0.2958	0.037*	
C5	0.28706 (17)	0.97857 (12)	0.33252 (7)	0.0295 (5)	
H5A	0.2639	0.9736	0.3610	0.035*	
C6	0.38890 (16)	0.97376 (11)	0.32733 (7)	0.0236 (4)	
C7	0.55559 (15)	0.96714 (10)	0.33578 (6)	0.0211 (4)	
C8	0.66268 (16)	0.95911 (12)	0.35676 (7)	0.0270 (5)	
H8A	0.6813	0.9988	0.3789	0.032*	
H8B	0.6735	0.9109	0.3720	0.032*	
C9	0.81783 (16)	0.93253 (12)	0.32635 (7)	0.0261 (5)	
H9A	0.8166	0.8835	0.3407	0.031*	
H9B	0.8663	0.9645	0.3451	0.031*	
C10	0.84499 (15)	0.92633 (11)	0.27992 (7)	0.0233 (4)	
C11	0.92802 (16)	0.90006 (11)	0.22272 (7)	0.0268 (5)	
C12	0.99580 (19)	0.87888 (14)	0.19389 (8)	0.0380 (6)	
H12A	1.0573	0.8545	0.2045	0.046*	
C13	0.9696 (2)	0.89491 (15)	0.14870 (8)	0.0448 (6)	
H13A	1.0144	0.8818	0.1278	0.054*	
C14	0.8783 (2)	0.93001 (15)	0.13348 (8)	0.0446 (6)	
H14A	0.8619	0.9392	0.1023	0.054*	
C15	0.81116 (19)	0.95166 (13)	0.16244 (7)	0.0341 (5)	
H15A	0.7495	0.9758	0.1518	0.041*	
C16	0.83743 (16)	0.93669 (11)	0.20773 (7)	0.0263 (5)	
C17	0.47390 (18)	0.94909 (12)	0.40680 (7)	0.0281 (5)	
H17A	0.5410	0.9300	0.4196	0.034*	
H17B	0.4232	0.9104	0.4104	0.034*	
C18	0.44972 (16)	1.01602 (12)	0.43293 (7)	0.0253 (4)	
C19	0.4996 (2)	1.08176 (13)	0.42855 (8)	0.0364 (5)	
H19A	0.5472	1.0854	0.4076	0.044*	
C20	0.4813 (2)	1.14228 (14)	0.45412 (8)	0.0457 (7)	
H20A	0.5174	1.1868	0.4512	0.055*	
C21	0.4112 (2)	1.13830 (15)	0.48382 (8)	0.0457 (7)	
H21A	0.3974	1.1803	0.5009	0.055*	
C22	0.36073 (19)	1.07298 (17)	0.48872 (8)	0.0433 (6)	
H22A	0.3123	1.0699	0.5093	0.052*	
C23	0.38073 (18)	1.01153 (14)	0.46346 (8)	0.0353 (5)	
H23A	0.3468	0.9664	0.4673	0.042*	
C24	1.00640 (16)	0.85285 (12)	0.29916 (7)	0.0283 (5)	
H24A	0.9989	0.8657	0.3306	0.034*	
H24B	1.0747	0.8687	0.2934	0.034*	
C25	0.99832 (15)	0.77029 (12)	0.29365 (7)	0.0252 (4)	
C26	0.91468 (17)	0.73262 (13)	0.30530 (8)	0.0314 (5)	
H26A	0.8608	0.7593	0.3156	0.038*	
C27	0.90888 (18)	0.65670 (13)	0.30215 (8)	0.0355 (5)	
H27A	0.8511	0.6317	0.3101	0.043*	
C28	0.98740 (19)	0.61710 (13)	0.28745 (8)	0.0370 (6)	
H28A	0.9839	0.5649	0.2856	0.044*	
C29	1.07080 (19)	0.65395 (13)	0.27557 (8)	0.0366 (5)	
H29A	1.1248	0.6271	0.2655	0.044*	
C30	1.07551 (17)	0.72998 (12)	0.27840 (7)	0.0302 (5)	
H30A	1.1326	0.7550	0.2697	0.036*	
C31	0.58910 (15)	0.82256 (11)	0.21604 (7)	0.0222 (4)	
C32	0.63884 (16)	0.78461 (12)	0.25259 (7)	0.0277 (5)	
H32A	0.6757	0.8099	0.2771	0.033*	
C33	0.63265 (18)	0.70887 (12)	0.25184 (8)	0.0331 (5)	
H33A	0.6661	0.6814	0.2762	0.040*	
C34	0.57790 (18)	0.67173 (12)	0.21575 (8)	0.0347 (5)	
H34A	0.5748	0.6195	0.2164	0.042*	
C35	0.52805 (18)	0.70878 (12)	0.17908 (8)	0.0309 (5)	
H35A	0.4907	0.6835	0.1547	0.037*	
C36	0.53579 (16)	0.78486 (11)	0.17999 (7)	0.0248 (4)	
C37	0.52721 (15)	0.90414 (11)	0.16806 (7)	0.0216 (4)	
C38	0.50469 (17)	0.97679 (11)	0.14638 (7)	0.0266 (5)	
H38A	0.5411	0.9830	0.1198	0.032*	
H38B	0.4312	0.9829	0.1370	0.032*	
C39	0.53686 (17)	1.10210 (10)	0.16844 (7)	0.0250 (4)	
H39A	0.4662	1.1201	0.1630	0.030*	
H39B	0.5697	1.1102	0.1410	0.030*	
C40	0.59380 (15)	1.13949 (11)	0.20783 (7)	0.0219 (4)	
C41	0.65688 (15)	1.22920 (11)	0.25217 (7)	0.0233 (4)	
C42	0.68580 (17)	1.29543 (12)	0.27277 (8)	0.0296 (5)	
H42A	0.6663	1.3414	0.2592	0.035*	
C43	0.74447 (17)	1.29117 (12)	0.31400 (8)	0.0329 (5)	
H43A	0.7654	1.3352	0.3295	0.039*	
C44	0.77373 (17)	1.22329 (13)	0.33333 (8)	0.0331 (5)	
H44A	0.8144	1.2225	0.3617	0.040*	
C45	0.74529 (16)	1.15698 (12)	0.31241 (7)	0.0283 (5)	
H45A	0.7657	1.1111	0.3258	0.034*	
C46	0.68584 (15)	1.16080 (11)	0.27114 (7)	0.0229 (4)	
C47	0.43576 (17)	0.82781 (12)	0.10608 (7)	0.0290 (5)	
H47A	0.3724	0.8566	0.1050	0.035*	
H47B	0.4171	0.7752	0.1026	0.035*	
C48	0.49104 (17)	0.85072 (11)	0.06750 (7)	0.0282 (5)	
C49	0.5921 (2)	0.83515 (15)	0.06703 (9)	0.0444 (6)	
H49A	0.6287	0.8102	0.0918	0.053*	
C50	0.6408 (2)	0.85553 (18)	0.03091 (10)	0.0568 (8)	
H50A	0.7106	0.8446	0.0311	0.068*	
C51	0.5896 (2)	0.89111 (16)	−0.00503 (9)	0.0519 (7)	
H51A	0.6234	0.9051	−0.0297	0.062*	
C52	0.4890 (2)	0.90652 (18)	−0.00514 (9)	0.0539 (8)	
H52A	0.4528	0.9310	−0.0302	0.065*	
C53	0.4395 (2)	0.88672 (15)	0.03101 (8)	0.0415 (6)	
H53A	0.3698	0.8980	0.0307	0.050*	
C54	0.54907 (17)	1.26622 (11)	0.17870 (7)	0.0274 (5)	
H54A	0.5462	1.3150	0.1932	0.033*	
H54B	0.4787	1.2501	0.1689	0.033*	
C55	0.60443 (17)	1.27339 (11)	0.13814 (7)	0.0271 (5)	
C56	0.70172 (18)	1.30224 (12)	0.14248 (8)	0.0347 (5)	
H56A	0.7340	1.3163	0.1713	0.042*	
C57	0.7517 (2)	1.31053 (14)	0.10528 (10)	0.0474 (7)	
H57A	0.8179	1.3310	0.1084	0.057*	
C58	0.7055 (2)	1.28898 (15)	0.06341 (9)	0.0495 (7)	
H58A	0.7402	1.2945	0.0378	0.059*	
C59	0.6098 (2)	1.25962 (14)	0.05872 (8)	0.0435 (6)	
H59A	0.5785	1.2447	0.0299	0.052*	
C60	0.55879 (19)	1.25165 (12)	0.09604 (7)	0.0329 (5)	
H60A	0.4926	1.2313	0.0927	0.039*	
C61	0.75450 (16)	0.87863 (13)	−0.10704 (7)	0.0311 (5)	
C62	0.80654 (17)	0.94103 (12)	−0.08451 (7)	0.0294 (5)	
C63	0.86873 (16)	0.93764 (13)	−0.04457 (7)	0.0290 (5)	
H63A	0.8982	0.9811	−0.0311	0.035*	
C64	0.88791 (16)	0.86988 (12)	−0.02421 (7)	0.0271 (5)	
C65	0.84019 (16)	0.80636 (13)	−0.04190 (7)	0.0290 (5)	
H65A	0.8507	0.7605	−0.0268	0.035*	
C66	0.77756 (16)	0.81147 (13)	−0.08177 (7)	0.0300 (5)	
C67	1.07224 (18)	0.58171 (12)	0.07767 (7)	0.0305 (5)	
C68	1.11109 (17)	0.62750 (13)	0.04350 (7)	0.0313 (5)	
C69	1.06011 (18)	0.64184 (13)	0.00217 (7)	0.0324 (5)	
H69A	1.0877	0.6745	−0.0178	0.039*	
C70	0.96760 (18)	0.60822 (12)	−0.01038 (7)	0.0296 (5)	
C71	0.92477 (17)	0.56267 (12)	0.01889 (7)	0.0300 (5)	
H71A	0.8616	0.5396	0.0097	0.036*	
C72	0.97367 (17)	0.55091 (12)	0.06127 (7)	0.0281 (5)	
C73	0.75874 (19)	0.11133 (13)	0.10514 (8)	0.0349 (5)	
H73A	0.7651	0.1372	0.1329	0.042*	
C74	0.8282 (2)	0.08428 (17)	0.03657 (8)	0.0492 (7)	
H74A	0.7654	0.0559	0.0306	0.074*	
H74B	0.8863	0.0511	0.0368	0.074*	
H74C	0.8304	0.1214	0.0130	0.074*	
C75	0.9203 (2)	0.16543 (16)	0.09386 (9)	0.0465 (6)	
H75A	0.9143	0.1871	0.1233	0.070*	
H75B	0.9249	0.2048	0.0719	0.070*	
H75C	0.9813	0.1348	0.0959	0.070*	
C76	0.8832 (2)	0.72248 (16)	0.09019 (9)	0.0515 (7)	
H76A	0.9520	0.7353	0.0891	0.062*	
C77	0.7586 (3)	0.6747 (3)	0.13267 (16)	0.1046 (17)	
H77A	0.7158	0.6842	0.1042	0.157*	
H77B	0.7351	0.7046	0.1565	0.157*	
H77C	0.7547	0.6224	0.1404	0.157*	
C78	0.9360 (2)	0.68289 (18)	0.16708 (9)	0.0561 (8)	
H78A	1.0026	0.6972	0.1595	0.084*	
H78B	0.9373	0.6310	0.1760	0.084*	
H78C	0.9187	0.7134	0.1920	0.084*	

### Atomic displacement parameters (Å^2^)

**Table:**

	*U*^11^	*U*^22^	*U*^33^	*U*^12^	*U*^13^	*U*^23^
Zn	0.02450 (13)	0.01631 (12)	0.01856 (12)	0.00111 (9)	0.00018 (9)	0.00033 (9)
O1	0.0264 (8)	0.0458 (9)	0.0218 (7)	0.0124 (7)	0.0040 (6)	0.0057 (7)
O2	0.0528 (10)	0.0166 (7)	0.0226 (7)	−0.0046 (7)	−0.0045 (7)	0.0016 (6)
O3	0.0338 (9)	0.0574 (11)	0.0316 (9)	0.0018 (8)	−0.0076 (7)	0.0001 (8)
O4	0.129 (2)	0.0533 (13)	0.0301 (10)	−0.0022 (13)	−0.0038 (12)	0.0101 (9)
O5	0.0720 (14)	0.0432 (11)	0.0544 (12)	0.0265 (10)	0.0031 (10)	−0.0019 (10)
O6	0.0460 (10)	0.0505 (11)	0.0266 (8)	−0.0021 (8)	−0.0022 (7)	−0.0066 (8)
O7	0.0463 (11)	0.0431 (10)	0.0472 (10)	0.0030 (8)	−0.0096 (9)	0.0121 (8)
O8	0.0605 (12)	0.0477 (11)	0.0450 (10)	−0.0182 (9)	0.0139 (9)	−0.0015 (9)
O9	0.0435 (10)	0.0541 (11)	0.0316 (9)	−0.0091 (8)	0.0029 (8)	−0.0122 (8)
O10	0.0508 (11)	0.0529 (11)	0.0287 (8)	−0.0126 (9)	−0.0122 (8)	0.0100 (8)
O11	0.0347 (11)	0.1035 (18)	0.0757 (15)	−0.0012 (11)	−0.0049 (11)	0.0239 (14)
O12	0.0586 (13)	0.0581 (13)	0.0550 (12)	−0.0234 (10)	0.0108 (10)	−0.0014 (10)
O13	0.0759 (14)	0.0499 (11)	0.0269 (9)	0.0046 (10)	0.0000 (9)	0.0096 (8)
O14	0.0495 (12)	0.0763 (14)	0.0318 (9)	−0.0010 (10)	−0.0124 (9)	−0.0029 (9)
O15	0.0534 (12)	0.0500 (11)	0.0509 (11)	−0.0184 (9)	0.0057 (9)	0.0031 (9)
O16	0.0498 (12)	0.0695 (13)	0.0323 (9)	−0.0053 (9)	0.0045 (9)	0.0165 (9)
O17	0.0411 (10)	0.0422 (10)	0.0422 (10)	0.0029 (8)	0.0071 (8)	0.0070 (8)
O18	0.0608 (14)	0.111 (2)	0.0582 (13)	0.0030 (13)	−0.0056 (11)	0.0370 (14)
N1	0.0321 (10)	0.0154 (8)	0.0183 (8)	0.0019 (7)	−0.0016 (7)	0.0000 (6)
N2	0.0305 (10)	0.0218 (8)	0.0184 (8)	0.0022 (7)	0.0037 (7)	0.0006 (7)
N3	0.0231 (9)	0.0211 (8)	0.0228 (8)	0.0032 (7)	0.0010 (7)	0.0002 (7)
N4	0.0243 (9)	0.0256 (9)	0.0243 (9)	0.0049 (7)	0.0006 (7)	−0.0002 (7)
N5	0.0284 (9)	0.0189 (8)	0.0189 (8)	−0.0010 (7)	0.0012 (7)	−0.0001 (7)
N6	0.0291 (9)	0.0218 (9)	0.0219 (8)	−0.0018 (7)	0.0020 (7)	−0.0020 (7)
N7	0.0246 (9)	0.0182 (8)	0.0221 (8)	0.0013 (7)	0.0023 (7)	0.0004 (7)
N8	0.0291 (9)	0.0160 (8)	0.0245 (9)	0.0018 (7)	0.0034 (7)	0.0029 (7)
N9	0.0386 (12)	0.0416 (12)	0.0361 (11)	0.0058 (9)	−0.0029 (9)	0.0026 (10)
N10	0.0325 (10)	0.0410 (12)	0.0245 (9)	−0.0010 (9)	0.0028 (8)	0.0022 (9)
N11	0.0290 (10)	0.0437 (12)	0.0322 (10)	−0.0030 (9)	0.0077 (8)	−0.0056 (9)
N12	0.0373 (12)	0.0607 (15)	0.0307 (11)	−0.0063 (11)	0.0076 (9)	−0.0013 (10)
N13	0.0498 (13)	0.0414 (12)	0.0249 (10)	0.0131 (10)	−0.0025 (9)	−0.0001 (9)
N14	0.0331 (11)	0.0343 (11)	0.0348 (11)	0.0046 (8)	0.0048 (9)	0.0047 (9)
N15	0.0362 (11)	0.0362 (11)	0.0273 (10)	0.0048 (9)	0.0063 (8)	−0.0021 (8)
N16	0.0416 (12)	0.0477 (13)	0.0398 (12)	0.0076 (10)	0.0070 (10)	0.0092 (10)
C1	0.0240 (10)	0.0137 (9)	0.0256 (10)	−0.0007 (8)	0.0012 (8)	−0.0011 (8)
C2	0.0259 (11)	0.0192 (9)	0.0238 (10)	−0.0012 (8)	0.0024 (9)	0.0003 (8)
C3	0.0328 (12)	0.0227 (11)	0.0291 (11)	0.0003 (9)	−0.0046 (9)	−0.0008 (9)
C4	0.0259 (11)	0.0297 (11)	0.0367 (12)	0.0000 (9)	0.0032 (10)	0.0002 (10)
C5	0.0351 (12)	0.0269 (11)	0.0279 (11)	0.0006 (9)	0.0097 (9)	0.0008 (9)
C6	0.0295 (11)	0.0176 (10)	0.0235 (10)	0.0009 (8)	0.0030 (9)	0.0002 (8)
C7	0.0268 (11)	0.0161 (9)	0.0208 (9)	0.0015 (8)	0.0044 (8)	0.0006 (8)
C8	0.0320 (12)	0.0299 (11)	0.0188 (10)	0.0052 (9)	0.0014 (9)	−0.0029 (9)
C9	0.0252 (11)	0.0289 (11)	0.0230 (10)	0.0079 (9)	−0.0019 (9)	−0.0017 (9)
C10	0.0245 (10)	0.0185 (10)	0.0257 (10)	0.0003 (8)	−0.0019 (8)	−0.0023 (8)
C11	0.0293 (11)	0.0254 (11)	0.0261 (10)	0.0037 (9)	0.0054 (9)	0.0025 (9)
C12	0.0380 (14)	0.0401 (13)	0.0368 (13)	0.0115 (11)	0.0080 (11)	0.0048 (11)
C13	0.0491 (16)	0.0534 (16)	0.0345 (13)	0.0137 (13)	0.0160 (12)	0.0061 (12)
C14	0.0513 (16)	0.0555 (16)	0.0287 (12)	0.0134 (13)	0.0114 (11)	0.0105 (12)
C15	0.0392 (13)	0.0359 (13)	0.0270 (11)	0.0082 (10)	0.0039 (10)	0.0075 (10)
C16	0.0321 (12)	0.0222 (10)	0.0252 (10)	0.0034 (9)	0.0052 (9)	0.0017 (8)
C17	0.0397 (13)	0.0260 (11)	0.0188 (10)	0.0013 (9)	0.0040 (9)	0.0033 (8)
C18	0.0285 (11)	0.0300 (11)	0.0169 (9)	−0.0001 (9)	0.0009 (8)	0.0014 (8)
C19	0.0520 (15)	0.0314 (12)	0.0269 (11)	−0.0031 (11)	0.0096 (11)	0.0001 (10)
C20	0.0719 (19)	0.0318 (13)	0.0346 (13)	−0.0030 (13)	0.0120 (13)	−0.0065 (11)
C21	0.0598 (18)	0.0421 (15)	0.0346 (13)	0.0105 (13)	0.0034 (13)	−0.0143 (11)
C22	0.0340 (14)	0.0697 (19)	0.0272 (12)	0.0026 (13)	0.0070 (10)	−0.0159 (12)
C23	0.0315 (12)	0.0485 (14)	0.0261 (11)	−0.0100 (11)	0.0041 (10)	−0.0056 (10)
C24	0.0236 (11)	0.0284 (11)	0.0309 (11)	0.0037 (9)	−0.0044 (9)	0.0000 (9)
C25	0.0224 (10)	0.0282 (11)	0.0243 (10)	0.0045 (8)	−0.0002 (8)	0.0026 (9)
C26	0.0263 (11)	0.0334 (12)	0.0350 (12)	0.0042 (9)	0.0054 (10)	−0.0006 (10)
C27	0.0324 (13)	0.0344 (13)	0.0409 (13)	−0.0019 (10)	0.0093 (11)	0.0037 (10)
C28	0.0417 (14)	0.0275 (12)	0.0420 (13)	0.0015 (10)	0.0052 (11)	−0.0013 (10)
C29	0.0349 (13)	0.0338 (13)	0.0425 (13)	0.0080 (10)	0.0100 (11)	−0.0029 (11)
C30	0.0277 (11)	0.0335 (12)	0.0299 (11)	0.0023 (9)	0.0056 (9)	0.0016 (10)
C31	0.0256 (11)	0.0194 (10)	0.0224 (10)	−0.0001 (8)	0.0067 (8)	−0.0004 (8)
C32	0.0297 (12)	0.0260 (11)	0.0274 (11)	0.0005 (9)	0.0041 (9)	0.0013 (9)
C33	0.0375 (13)	0.0268 (11)	0.0357 (12)	0.0062 (10)	0.0078 (10)	0.0098 (10)
C34	0.0434 (14)	0.0190 (10)	0.0435 (13)	0.0002 (10)	0.0121 (11)	0.0025 (10)
C35	0.0388 (13)	0.0224 (11)	0.0330 (12)	−0.0036 (9)	0.0100 (10)	−0.0056 (9)
C36	0.0278 (11)	0.0226 (10)	0.0252 (10)	−0.0002 (8)	0.0080 (9)	−0.0013 (8)
C37	0.0215 (10)	0.0210 (10)	0.0225 (10)	−0.0032 (8)	0.0033 (8)	−0.0027 (8)
C38	0.0372 (12)	0.0203 (10)	0.0202 (10)	−0.0023 (9)	−0.0043 (9)	−0.0021 (8)
C39	0.0315 (11)	0.0175 (10)	0.0253 (10)	0.0005 (8)	0.0008 (9)	0.0041 (8)
C40	0.0238 (10)	0.0192 (10)	0.0235 (10)	0.0006 (8)	0.0057 (8)	0.0035 (8)
C41	0.0248 (10)	0.0211 (10)	0.0257 (10)	−0.0001 (8)	0.0096 (8)	0.0004 (8)
C42	0.0331 (12)	0.0208 (10)	0.0362 (12)	−0.0025 (9)	0.0098 (10)	−0.0010 (9)
C43	0.0351 (13)	0.0288 (12)	0.0363 (12)	−0.0074 (10)	0.0105 (10)	−0.0114 (10)
C44	0.0308 (12)	0.0370 (13)	0.0308 (12)	−0.0049 (10)	0.0006 (10)	−0.0050 (10)
C45	0.0295 (11)	0.0270 (11)	0.0280 (11)	−0.0005 (9)	0.0020 (9)	0.0022 (9)
C46	0.0246 (10)	0.0195 (10)	0.0252 (10)	−0.0002 (8)	0.0056 (8)	−0.0005 (8)
C47	0.0340 (12)	0.0303 (11)	0.0218 (10)	−0.0061 (9)	0.0000 (9)	−0.0059 (9)
C48	0.0363 (12)	0.0246 (11)	0.0238 (10)	−0.0039 (9)	0.0040 (9)	−0.0070 (9)
C49	0.0436 (15)	0.0520 (16)	0.0393 (14)	0.0124 (12)	0.0116 (12)	0.0091 (12)
C50	0.0453 (17)	0.073 (2)	0.0565 (18)	0.0096 (15)	0.0234 (14)	0.0086 (16)
C51	0.064 (2)	0.0597 (18)	0.0362 (14)	−0.0078 (15)	0.0234 (14)	0.0009 (13)
C52	0.066 (2)	0.0651 (19)	0.0297 (13)	−0.0015 (15)	0.0030 (13)	0.0097 (13)
C53	0.0413 (14)	0.0546 (16)	0.0268 (12)	−0.0019 (12)	−0.0026 (11)	0.0018 (11)
C54	0.0345 (12)	0.0189 (10)	0.0289 (11)	0.0074 (9)	0.0046 (9)	0.0073 (9)
C55	0.0332 (12)	0.0181 (10)	0.0312 (11)	0.0051 (9)	0.0080 (9)	0.0080 (9)
C56	0.0385 (13)	0.0260 (11)	0.0392 (13)	0.0010 (10)	0.0034 (11)	0.0089 (10)
C57	0.0444 (15)	0.0387 (14)	0.0626 (18)	0.0024 (12)	0.0197 (14)	0.0162 (13)
C58	0.0636 (19)	0.0460 (16)	0.0440 (15)	0.0157 (14)	0.0269 (14)	0.0154 (13)
C59	0.0640 (18)	0.0351 (13)	0.0316 (13)	0.0142 (13)	0.0069 (12)	0.0051 (11)
C60	0.0411 (13)	0.0259 (11)	0.0311 (11)	0.0059 (10)	0.0025 (10)	0.0054 (9)
C61	0.0240 (11)	0.0455 (13)	0.0242 (11)	0.0030 (10)	0.0048 (9)	−0.0030 (10)
C62	0.0290 (11)	0.0347 (12)	0.0251 (11)	0.0046 (9)	0.0054 (9)	0.0014 (9)
C63	0.0290 (11)	0.0333 (12)	0.0258 (10)	0.0030 (9)	0.0073 (9)	−0.0041 (9)
C64	0.0262 (11)	0.0360 (12)	0.0195 (10)	0.0020 (9)	0.0039 (9)	−0.0021 (9)
C65	0.0268 (11)	0.0350 (12)	0.0267 (11)	0.0012 (9)	0.0087 (9)	0.0003 (9)
C66	0.0260 (11)	0.0365 (12)	0.0279 (11)	−0.0029 (9)	0.0049 (9)	−0.0039 (10)
C67	0.0378 (13)	0.0286 (11)	0.0244 (10)	0.0019 (10)	0.0011 (10)	0.0007 (9)
C68	0.0307 (12)	0.0352 (12)	0.0273 (11)	0.0018 (10)	0.0012 (9)	−0.0011 (10)
C69	0.0406 (13)	0.0304 (12)	0.0267 (11)	0.0026 (10)	0.0058 (10)	0.0009 (9)
C70	0.0377 (13)	0.0305 (12)	0.0195 (10)	0.0078 (10)	−0.0005 (9)	−0.0004 (9)
C71	0.0314 (12)	0.0287 (11)	0.0288 (11)	0.0060 (9)	−0.0003 (9)	−0.0052 (9)
C72	0.0315 (12)	0.0261 (11)	0.0269 (11)	0.0018 (9)	0.0044 (9)	0.0028 (9)
C73	0.0388 (14)	0.0360 (13)	0.0310 (12)	0.0110 (11)	0.0089 (11)	0.0037 (10)
C74	0.0451 (16)	0.0676 (19)	0.0355 (13)	0.0022 (14)	0.0079 (12)	−0.0147 (13)
C75	0.0414 (15)	0.0531 (16)	0.0456 (15)	0.0001 (12)	0.0083 (12)	−0.0095 (13)
C76	0.0499 (17)	0.0576 (18)	0.0472 (16)	0.0064 (14)	0.0072 (14)	0.0097 (14)
C77	0.052 (2)	0.143 (4)	0.117 (3)	−0.011 (2)	0.004 (2)	0.079 (3)
C78	0.0624 (19)	0.0607 (19)	0.0432 (15)	0.0029 (15)	−0.0012 (14)	0.0041 (14)

### Geometric parameters (Å, °)

**Table:**

Zn—N1	2.0974 (18)	C24—C25	1.511 (3)
Zn—N3	2.0988 (17)	C24—H24A	0.9900
Zn—N7	2.1106 (16)	C24—H24B	0.9900
Zn—N5	2.1164 (16)	C25—C30	1.387 (3)
Zn—O2	2.3533 (14)	C25—C26	1.389 (3)
Zn—O1	2.3567 (14)	C26—C27	1.384 (3)
O1—C9	1.420 (2)	C26—H26A	0.9500
O1—C8	1.417 (3)	C27—C28	1.387 (3)
O2—C39	1.409 (2)	C27—H27A	0.9500
O2—C38	1.412 (2)	C28—C29	1.382 (4)
O3—C61	1.244 (3)	C28—H28A	0.9500
O4—N9	1.215 (3)	C29—C30	1.384 (3)
O5—N9	1.222 (3)	C29—H29A	0.9500
O6—N10	1.233 (2)	C30—H30A	0.9500
O7—N10	1.226 (3)	C31—C32	1.391 (3)
O8—N11	1.227 (3)	C31—C36	1.396 (3)
O9—N11	1.233 (2)	C32—C33	1.378 (3)
O10—C67	1.234 (2)	C32—H32A	0.9500
O11—N12	1.220 (3)	C33—C34	1.400 (3)
O12—N12	1.217 (3)	C33—H33A	0.9500
O13—N13	1.239 (3)	C34—C35	1.386 (3)
O14—N13	1.234 (3)	C34—H34A	0.9500
O15—N14	1.232 (3)	C35—C36	1.386 (3)
O16—N14	1.226 (3)	C35—H35A	0.9500
O17—C73	1.227 (3)	C37—C38	1.485 (3)
O18—C76	1.211 (3)	C38—H38A	0.9900
N1—C7	1.324 (2)	C38—H38B	0.9900
N1—C1	1.396 (3)	C39—C40	1.486 (3)
N2—C7	1.347 (3)	C39—H39A	0.9900
N2—C6	1.396 (3)	C39—H39B	0.9900
N2—C17	1.474 (2)	C41—C42	1.385 (3)
N3—C10	1.322 (2)	C41—C46	1.400 (3)
N3—C16	1.394 (3)	C42—C43	1.380 (3)
N4—C10	1.358 (3)	C42—H42A	0.9500
N4—C11	1.391 (3)	C43—C44	1.397 (3)
N4—C24	1.472 (3)	C43—H43A	0.9500
N5—C37	1.322 (2)	C44—C45	1.389 (3)
N5—C31	1.390 (2)	C44—H44A	0.9500
N6—C37	1.350 (2)	C45—C46	1.385 (3)
N6—C36	1.390 (3)	C45—H45A	0.9500
N6—C47	1.465 (2)	C47—C48	1.507 (3)
N7—C40	1.321 (2)	C47—H47A	0.9900
N7—C46	1.407 (3)	C47—H47B	0.9900
N8—C40	1.347 (2)	C48—C49	1.378 (3)
N8—C41	1.392 (3)	C48—C53	1.381 (3)
N8—C54	1.470 (2)	C49—C50	1.382 (4)
N9—C62	1.456 (3)	C49—H49A	0.9500
N10—C64	1.443 (3)	C50—C51	1.364 (4)
N11—C66	1.457 (3)	C50—H50A	0.9500
N12—C68	1.454 (3)	C51—C52	1.369 (4)
N13—C70	1.441 (3)	C51—H51A	0.9500
N14—C72	1.456 (3)	C52—C53	1.386 (4)
N15—C73	1.322 (3)	C52—H52A	0.9500
N15—C75	1.450 (3)	C53—H53A	0.9500
N15—C74	1.449 (3)	C54—C55	1.507 (3)
N16—C76	1.326 (3)	C54—H54A	0.9900
N16—C77	1.423 (4)	C54—H54B	0.9900
N16—C78	1.453 (3)	C55—C56	1.389 (3)
C1—C2	1.400 (3)	C55—C60	1.389 (3)
C1—C6	1.405 (3)	C56—C57	1.379 (4)
C2—C3	1.372 (3)	C56—H56A	0.9500
C2—H2A	0.9500	C57—C58	1.384 (4)
C3—C4	1.403 (3)	C57—H57A	0.9500
C3—H3A	0.9500	C58—C59	1.373 (4)
C4—C5	1.384 (3)	C58—H58A	0.9500
C4—H4A	0.9500	C59—C60	1.389 (4)
C5—C6	1.388 (3)	C59—H59A	0.9500
C5—H5A	0.9500	C60—H60A	0.9500
C7—C8	1.492 (3)	C61—C66	1.449 (3)
C8—H8A	0.9900	C61—C62	1.450 (3)
C8—H8B	0.9900	C62—C63	1.370 (3)
C9—C10	1.488 (3)	C63—C64	1.384 (3)
C9—H9A	0.9900	C63—H63A	0.9500
C9—H9B	0.9900	C64—C65	1.390 (3)
C11—C12	1.382 (3)	C65—C66	1.373 (3)
C11—C16	1.402 (3)	C65—H65A	0.9500
C12—C13	1.388 (3)	C67—C72	1.455 (3)
C12—H12A	0.9500	C67—C68	1.465 (3)
C13—C14	1.398 (4)	C68—C69	1.362 (3)
C13—H13A	0.9500	C69—C70	1.384 (3)
C14—C15	1.381 (3)	C69—H69A	0.9500
C14—H14A	0.9500	C70—C71	1.382 (3)
C15—C16	1.387 (3)	C71—C72	1.371 (3)
C15—H15A	0.9500	C71—H71A	0.9500
C17—C18	1.504 (3)	C73—H73A	0.9500
C17—H17A	0.9900	C74—H74A	0.9800
C17—H17B	0.9900	C74—H74B	0.9800
C18—C19	1.381 (3)	C74—H74C	0.9800
C18—C23	1.380 (3)	C75—H75A	0.9800
C19—C20	1.380 (3)	C75—H75B	0.9800
C19—H19A	0.9500	C75—H75C	0.9800
C20—C21	1.373 (4)	C76—H76A	0.9500
C20—H20A	0.9500	C77—H77A	0.9800
C21—C22	1.381 (4)	C77—H77B	0.9800
C21—H21A	0.9500	C77—H77C	0.9800
C22—C23	1.394 (4)	C78—H78A	0.9800
C22—H22A	0.9500	C78—H78B	0.9800
C23—H23A	0.9500	C78—H78C	0.9800
			
N1—Zn—N3	136.28 (6)	C29—C30—H30A	119.4
N1—Zn—N7	100.17 (6)	N5—C31—C32	130.01 (18)
N3—Zn—N7	105.03 (7)	N5—C31—C36	109.11 (17)
N1—Zn—N5	93.77 (6)	C32—C31—C36	120.88 (19)
N3—Zn—N5	90.27 (7)	C33—C32—C31	117.4 (2)
N7—Zn—N5	138.74 (6)	C33—C32—H32A	121.3
N1—Zn—O2	102.74 (6)	C31—C32—H32A	121.3
N3—Zn—O2	119.08 (6)	C32—C33—C34	121.2 (2)
N7—Zn—O2	70.51 (5)	C32—C33—H33A	119.4
N5—Zn—O2	68.58 (5)	C34—C33—H33A	119.4
N1—Zn—O1	70.02 (6)	C35—C34—C33	122.1 (2)
N3—Zn—O1	69.62 (6)	C35—C34—H34A	119.0
N7—Zn—O1	103.72 (6)	C33—C34—H34A	119.0
N5—Zn—O1	117.55 (6)	C34—C35—C36	116.2 (2)
O2—Zn—O1	170.22 (5)	C34—C35—H35A	121.9
C9—O1—C8	116.46 (15)	C36—C35—H35A	121.9
C9—O1—Zn	120.42 (12)	C35—C36—N6	131.91 (19)
C8—O1—Zn	120.22 (11)	C35—C36—C31	122.2 (2)
C39—O2—C38	116.10 (15)	N6—C36—C31	105.83 (17)
C39—O2—Zn	120.11 (11)	N5—C37—N6	113.21 (17)
C38—O2—Zn	121.75 (12)	N5—C37—C38	122.02 (17)
C7—N1—C1	105.49 (17)	N6—C37—C38	124.75 (17)
C7—N1—Zn	122.89 (14)	O2—C38—C37	103.06 (15)
C1—N1—Zn	131.60 (13)	O2—C38—H38A	111.2
C7—N2—C6	107.08 (16)	C37—C38—H38A	111.2
C7—N2—C17	127.81 (17)	O2—C38—H38B	111.2
C6—N2—C17	125.09 (18)	C37—C38—H38B	111.2
C10—N3—C16	105.64 (17)	H38A—C38—H38B	109.1
C10—N3—Zn	122.61 (14)	O2—C39—C40	103.83 (15)
C16—N3—Zn	130.32 (13)	O2—C39—H39A	111.0
C10—N4—C11	106.96 (16)	C40—C39—H39A	111.0
C10—N4—C24	127.20 (18)	O2—C39—H39B	111.0
C11—N4—C24	125.60 (18)	C40—C39—H39B	111.0
C37—N5—C31	105.30 (16)	H39A—C39—H39B	109.0
C37—N5—Zn	123.62 (13)	N7—C40—N8	113.58 (17)
C31—N5—Zn	131.04 (13)	N7—C40—C39	124.14 (17)
C37—N6—C36	106.55 (16)	N8—C40—C39	122.28 (17)
C37—N6—C47	125.96 (17)	C42—C41—N8	131.64 (19)
C36—N6—C47	127.46 (17)	C42—C41—C46	122.83 (19)
C40—N7—C46	104.82 (16)	N8—C41—C46	105.53 (17)
C40—N7—Zn	120.92 (13)	C43—C42—C41	116.5 (2)
C46—N7—Zn	133.90 (13)	C43—C42—H42A	121.8
C40—N8—C41	107.03 (16)	C41—C42—H42A	121.8
C40—N8—C54	125.72 (17)	C42—C43—C44	121.2 (2)
C41—N8—C54	127.25 (17)	C42—C43—H43A	119.4
O4—N9—O5	122.9 (2)	C44—C43—H43A	119.4
O4—N9—C62	118.8 (2)	C45—C44—C43	122.1 (2)
O5—N9—C62	118.2 (2)	C45—C44—H44A	118.9
O7—N10—O6	123.45 (18)	C43—C44—H44A	118.9
O7—N10—C64	118.59 (19)	C46—C45—C44	116.97 (19)
O6—N10—C64	117.94 (19)	C46—C45—H45A	121.5
O8—N11—O9	123.0 (2)	C44—C45—H45A	121.5
O8—N11—C66	118.47 (19)	C45—C46—C41	120.34 (19)
O9—N11—C66	118.5 (2)	C45—C46—N7	130.63 (18)
O12—N12—O11	122.6 (2)	C41—C46—N7	109.03 (17)
O12—N12—C68	118.7 (2)	N6—C47—C48	112.66 (18)
O11—N12—C68	118.7 (2)	N6—C47—H47A	109.1
O14—N13—O13	123.07 (19)	C48—C47—H47A	109.1
O14—N13—C70	118.5 (2)	N6—C47—H47B	109.1
O13—N13—C70	118.4 (2)	C48—C47—H47B	109.1
O16—N14—O15	122.0 (2)	H47A—C47—H47B	107.8
O16—N14—C72	119.84 (19)	C49—C48—C53	118.5 (2)
O15—N14—C72	118.16 (19)	C49—C48—C47	121.6 (2)
C73—N15—C75	122.3 (2)	C53—C48—C47	119.9 (2)
C73—N15—C74	120.9 (2)	C48—C49—C50	120.6 (2)
C75—N15—C74	116.8 (2)	C48—C49—H49A	119.7
C76—N16—C77	120.0 (3)	C50—C49—H49A	119.7
C76—N16—C78	122.3 (3)	C51—C50—C49	120.6 (3)
C77—N16—C78	117.6 (3)	C51—C50—H50A	119.7
N1—C1—C2	131.6 (2)	C49—C50—H50A	119.7
N1—C1—C6	108.92 (17)	C50—C51—C52	119.4 (3)
C2—C1—C6	119.47 (19)	C50—C51—H51A	120.3
C3—C2—C1	117.9 (2)	C52—C51—H51A	120.3
C3—C2—H2A	121.0	C51—C52—C53	120.5 (3)
C1—C2—H2A	121.0	C51—C52—H52A	119.8
C2—C3—C4	121.7 (2)	C53—C52—H52A	119.8
C2—C3—H3A	119.1	C48—C53—C52	120.4 (3)
C4—C3—H3A	119.1	C48—C53—H53A	119.8
C5—C4—C3	121.6 (2)	C52—C53—H53A	119.8
C5—C4—H4A	119.2	N8—C54—C55	112.29 (17)
C3—C4—H4A	119.2	N8—C54—H54A	109.1
C4—C5—C6	116.2 (2)	C55—C54—H54A	109.1
C4—C5—H5A	121.9	N8—C54—H54B	109.1
C6—C5—H5A	121.9	C55—C54—H54B	109.1
C5—C6—N2	131.5 (2)	H54A—C54—H54B	107.9
C5—C6—C1	123.02 (19)	C56—C55—C60	119.3 (2)
N2—C6—C1	105.40 (18)	C56—C55—C54	120.2 (2)
N1—C7—N2	113.09 (17)	C60—C55—C54	120.5 (2)
N1—C7—C8	122.55 (19)	C57—C56—C55	120.4 (2)
N2—C7—C8	124.35 (17)	C57—C56—H56A	119.8
O1—C8—C7	104.16 (15)	C55—C56—H56A	119.8
O1—C8—H8A	110.9	C56—C57—C58	120.0 (3)
C7—C8—H8A	110.9	C56—C57—H57A	120.0
O1—C8—H8B	110.9	C58—C57—H57A	120.0
C7—C8—H8B	110.9	C59—C58—C57	120.2 (3)
H8A—C8—H8B	108.9	C59—C58—H58A	119.9
O1—C9—C10	103.69 (15)	C57—C58—H58A	119.9
O1—C9—H9A	111.0	C58—C59—C60	120.0 (2)
C10—C9—H9A	111.0	C58—C59—H59A	120.0
O1—C9—H9B	111.0	C60—C59—H59A	120.0
C10—C9—H9B	111.0	C59—C60—C55	120.1 (2)
H9A—C9—H9B	109.0	C59—C60—H60A	120.0
N3—C10—N4	112.73 (18)	C55—C60—H60A	120.0
N3—C10—C9	122.59 (18)	O3—C61—C66	125.1 (2)
N4—C10—C9	124.67 (17)	O3—C61—C62	123.9 (2)
C12—C11—N4	132.0 (2)	C66—C61—C62	110.90 (18)
C12—C11—C16	122.4 (2)	C63—C62—C61	125.0 (2)
N4—C11—C16	105.60 (18)	C63—C62—N9	116.7 (2)
C11—C12—C13	116.7 (2)	C61—C62—N9	118.23 (19)
C11—C12—H12A	121.6	C62—C63—C64	119.0 (2)
C13—C12—H12A	121.6	C62—C63—H63A	120.5
C12—C13—C14	121.1 (2)	C64—C63—H63A	120.5
C12—C13—H13A	119.5	C63—C64—C65	121.30 (19)
C14—C13—H13A	119.5	C63—C64—N10	119.04 (19)
C15—C14—C13	122.0 (2)	C65—C64—N10	119.6 (2)
C15—C14—H14A	119.0	C66—C65—C64	118.4 (2)
C13—C14—H14A	119.0	C66—C65—H65A	120.8
C14—C15—C16	117.2 (2)	C64—C65—H65A	120.8
C14—C15—H15A	121.4	C65—C66—C61	125.4 (2)
C16—C15—H15A	121.4	C65—C66—N11	116.5 (2)
C15—C16—N3	130.4 (2)	C61—C66—N11	117.96 (18)
C15—C16—C11	120.5 (2)	O10—C67—C72	125.2 (2)
N3—C16—C11	109.05 (17)	O10—C67—C68	123.3 (2)
N2—C17—C18	113.49 (17)	C72—C67—C68	111.43 (18)
N2—C17—H17A	108.9	C69—C68—N12	117.3 (2)
C18—C17—H17A	108.9	C69—C68—C67	124.5 (2)
N2—C17—H17B	108.9	N12—C68—C67	118.18 (19)
C18—C17—H17B	108.9	C68—C69—C70	119.2 (2)
H17A—C17—H17B	107.7	C68—C69—H69A	120.4
C19—C18—C23	118.9 (2)	C70—C69—H69A	120.4
C19—C18—C17	120.8 (2)	C71—C70—C69	121.07 (19)
C23—C18—C17	120.2 (2)	C71—C70—N13	118.8 (2)
C20—C19—C18	121.0 (2)	C69—C70—N13	120.0 (2)
C20—C19—H19A	119.5	C72—C71—C70	119.8 (2)
C18—C19—H19A	119.5	C72—C71—H71A	120.1
C21—C20—C19	120.1 (2)	C70—C71—H71A	120.1
C21—C20—H20A	119.9	C71—C72—N14	116.2 (2)
C19—C20—H20A	119.9	C71—C72—C67	123.8 (2)
C20—C21—C22	119.7 (2)	N14—C72—C67	119.92 (18)
C20—C21—H21A	120.2	O17—C73—N15	126.3 (2)
C22—C21—H21A	120.2	O17—C73—H73A	116.8
C21—C22—C23	120.1 (2)	N15—C73—H73A	116.8
C21—C22—H22A	120.0	N15—C74—H74A	109.5
C23—C22—H22A	120.0	N15—C74—H74B	109.5
C18—C23—C22	120.2 (2)	H74A—C74—H74B	109.5
C18—C23—H23A	119.9	N15—C74—H74C	109.5
C22—C23—H23A	119.9	H74A—C74—H74C	109.5
N4—C24—C25	113.52 (16)	H74B—C74—H74C	109.5
N4—C24—H24A	108.9	N15—C75—H75A	109.5
C25—C24—H24A	108.9	N15—C75—H75B	109.5
N4—C24—H24B	108.9	H75A—C75—H75B	109.5
C25—C24—H24B	108.9	N15—C75—H75C	109.5
H24A—C24—H24B	107.7	H75A—C75—H75C	109.5
C30—C25—C26	118.4 (2)	H75B—C75—H75C	109.5
C30—C25—C24	121.0 (2)	O18—C76—N16	126.2 (3)
C26—C25—C24	120.6 (2)	O18—C76—H76A	116.9
C27—C26—C25	120.9 (2)	N16—C76—H76A	116.9
C27—C26—H26A	119.6	N16—C77—H77A	109.5
C25—C26—H26A	119.6	N16—C77—H77B	109.5
C28—C27—C26	120.0 (2)	H77A—C77—H77B	109.5
C28—C27—H27A	120.0	N16—C77—H77C	109.5
C26—C27—H27A	120.0	H77A—C77—H77C	109.5
C29—C28—C27	119.6 (2)	H77B—C77—H77C	109.5
C29—C28—H28A	120.2	N16—C78—H78A	109.5
C27—C28—H28A	120.2	N16—C78—H78B	109.5
C28—C29—C30	119.9 (2)	H78A—C78—H78B	109.5
C28—C29—H29A	120.0	N16—C78—H78C	109.5
C30—C29—H29A	120.0	H78A—C78—H78C	109.5
C25—C30—C29	121.1 (2)	H78B—C78—H78C	109.5
C25—C30—H30A	119.4		
			
N1—Zn—O1—C9	156.43 (16)	C37—N5—C31—C36	−0.1 (2)
N3—Zn—O1—C9	−6.37 (15)	Zn—N5—C31—C36	177.52 (15)
N7—Zn—O1—C9	−107.57 (15)	N5—C31—C32—C33	−179.9 (2)
N5—Zn—O1—C9	72.79 (16)	C36—C31—C32—C33	−0.5 (3)
N1—Zn—O1—C8	−3.60 (14)	C31—C32—C33—C34	−0.3 (3)
N3—Zn—O1—C8	−166.40 (16)	C32—C33—C34—C35	0.4 (4)
N7—Zn—O1—C8	92.41 (15)	C33—C34—C35—C36	0.3 (4)
N5—Zn—O1—C8	−87.24 (15)	C34—C35—C36—N6	−179.6 (2)
N1—Zn—O2—C39	100.04 (16)	C34—C35—C36—C31	−1.1 (3)
N3—Zn—O2—C39	−93.16 (16)	C37—N6—C36—C35	179.4 (2)
N7—Zn—O2—C39	3.61 (15)	C47—N6—C36—C35	−2.7 (4)
N5—Zn—O2—C39	−170.90 (17)	C37—N6—C36—C31	0.8 (2)
N1—Zn—O2—C38	−96.86 (16)	C47—N6—C36—C31	178.6 (2)
N3—Zn—O2—C38	69.94 (17)	N5—C31—C36—C35	−179.2 (2)
N7—Zn—O2—C38	166.71 (18)	C32—C31—C36—C35	1.2 (3)
N5—Zn—O2—C38	−7.80 (16)	N5—C31—C36—N6	−0.4 (2)
N3—Zn—N1—C7	26.50 (19)	C32—C31—C36—N6	−179.94 (19)
N7—Zn—N1—C7	−98.16 (15)	C31—N5—C37—N6	0.7 (2)
N5—Zn—N1—C7	120.85 (15)	Zn—N5—C37—N6	−177.22 (14)
O2—Zn—N1—C7	−170.28 (14)	C31—N5—C37—C38	−178.33 (19)
O1—Zn—N1—C7	2.86 (14)	Zn—N5—C37—C38	3.8 (3)
N3—Zn—N1—C1	−155.33 (15)	C36—N6—C37—N5	−0.9 (2)
N7—Zn—N1—C1	80.02 (17)	C47—N6—C37—N5	−178.84 (19)
N5—Zn—N1—C1	−60.98 (17)	C36—N6—C37—C38	178.0 (2)
O2—Zn—N1—C1	7.89 (17)	C47—N6—C37—C38	0.1 (3)
O1—Zn—N1—C1	−178.97 (18)	C39—O2—C38—C37	174.49 (18)
N1—Zn—N3—C10	−14.3 (2)	Zn—O2—C38—C37	10.8 (2)
N7—Zn—N3—C10	108.75 (16)	N5—C37—C38—O2	−9.2 (3)
N5—Zn—N3—C10	−110.03 (16)	N6—C37—C38—O2	171.9 (2)
O2—Zn—N3—C10	−175.49 (14)	C38—O2—C39—C40	−170.54 (18)
O1—Zn—N3—C10	9.42 (15)	Zn—O2—C39—C40	−6.5 (2)
N1—Zn—N3—C16	150.02 (16)	C46—N7—C40—N8	0.0 (2)
N7—Zn—N3—C16	−86.95 (18)	Zn—N7—C40—N8	174.07 (13)
N5—Zn—N3—C16	54.28 (18)	C46—N7—C40—C39	−179.9 (2)
O2—Zn—N3—C16	−11.2 (2)	Zn—N7—C40—C39	−5.8 (3)
O1—Zn—N3—C16	173.72 (19)	C41—N8—C40—N7	0.0 (2)
N1—Zn—N5—C37	104.08 (17)	C54—N8—C40—N7	179.88 (19)
N3—Zn—N5—C37	−119.49 (17)	C41—N8—C40—C39	179.96 (19)
N7—Zn—N5—C37	−6.0 (2)	C54—N8—C40—C39	−0.2 (3)
O2—Zn—N5—C37	1.86 (16)	O2—C39—C40—N7	7.9 (3)
O1—Zn—N5—C37	173.48 (15)	O2—C39—C40—N8	−171.98 (19)
N1—Zn—N5—C31	−73.21 (18)	C40—N8—C41—C42	180.0 (2)
N3—Zn—N5—C31	63.22 (18)	C54—N8—C41—C42	0.1 (4)
N7—Zn—N5—C31	176.72 (16)	C40—N8—C41—C46	0.0 (2)
O2—Zn—N5—C31	−175.4 (2)	C54—N8—C41—C46	−179.88 (19)
O1—Zn—N5—C31	−3.8 (2)	N8—C41—C42—C43	179.1 (2)
N1—Zn—N7—C40	−98.91 (16)	C46—C41—C42—C43	−0.9 (3)
N3—Zn—N7—C40	117.15 (16)	C41—C42—C43—C44	0.8 (3)
N5—Zn—N7—C40	8.9 (2)	C42—C43—C44—C45	−0.3 (4)
O2—Zn—N7—C40	1.12 (15)	C43—C44—C45—C46	−0.1 (3)
O1—Zn—N7—C40	−170.64 (15)	C44—C45—C46—C41	0.0 (3)
N1—Zn—N7—C46	73.14 (19)	C44—C45—C46—N7	−179.4 (2)
N3—Zn—N7—C46	−70.8 (2)	C42—C41—C46—C45	0.5 (3)
N5—Zn—N7—C46	−179.06 (17)	N8—C41—C46—C45	−179.47 (19)
O2—Zn—N7—C46	173.2 (2)	C42—C41—C46—N7	−180.0 (2)
O1—Zn—N7—C46	1.4 (2)	N8—C41—C46—N7	0.0 (2)
C7—N1—C1—C2	179.1 (2)	C40—N7—C46—C45	179.4 (2)
Zn—N1—C1—C2	0.7 (3)	Zn—N7—C46—C45	6.5 (4)
C7—N1—C1—C6	0.9 (2)	C40—N7—C46—C41	0.0 (2)
Zn—N1—C1—C6	−177.48 (13)	Zn—N7—C46—C41	−172.97 (15)
N1—C1—C2—C3	−176.82 (19)	C37—N6—C47—C48	65.2 (3)
C6—C1—C2—C3	1.2 (3)	C36—N6—C47—C48	−112.3 (2)
C1—C2—C3—C4	0.1 (3)	N6—C47—C48—C49	41.1 (3)
C2—C3—C4—C5	−1.1 (3)	N6—C47—C48—C53	−140.4 (2)
C3—C4—C5—C6	0.7 (3)	C53—C48—C49—C50	0.3 (4)
C4—C5—C6—N2	178.4 (2)	C47—C48—C49—C50	178.8 (2)
C4—C5—C6—C1	0.7 (3)	C48—C49—C50—C51	−0.3 (5)
C7—N2—C6—C5	−176.6 (2)	C49—C50—C51—C52	−0.1 (5)
C17—N2—C6—C5	5.1 (3)	C50—C51—C52—C53	0.5 (5)
C7—N2—C6—C1	1.3 (2)	C49—C48—C53—C52	0.0 (4)
C17—N2—C6—C1	−176.92 (17)	C47—C48—C53—C52	−178.5 (2)
N1—C1—C6—C5	176.77 (18)	C51—C52—C53—C48	−0.4 (4)
C2—C1—C6—C5	−1.7 (3)	C40—N8—C54—C55	−75.7 (3)
N1—C1—C6—N2	−1.4 (2)	C41—N8—C54—C55	104.1 (2)
C2—C1—C6—N2	−179.86 (17)	N8—C54—C55—C56	−65.1 (2)
C1—N1—C7—N2	−0.1 (2)	N8—C54—C55—C60	115.2 (2)
Zn—N1—C7—N2	178.52 (12)	C60—C55—C56—C57	1.2 (3)
C1—N1—C7—C8	179.30 (18)	C54—C55—C56—C57	−178.4 (2)
Zn—N1—C7—C8	−2.1 (3)	C55—C56—C57—C58	−1.0 (4)
C6—N2—C7—N1	−0.8 (2)	C56—C57—C58—C59	0.3 (4)
C17—N2—C7—N1	177.36 (18)	C57—C58—C59—C60	0.2 (4)
C6—N2—C7—C8	179.82 (18)	C58—C59—C60—C55	0.0 (3)
C17—N2—C7—C8	−2.0 (3)	C56—C55—C60—C59	−0.8 (3)
C9—O1—C8—C7	−157.35 (17)	C54—C55—C60—C59	178.93 (19)
Zn—O1—C8—C7	3.4 (2)	O3—C61—C62—C63	175.5 (2)
N1—C7—C8—O1	−1.1 (3)	C66—C61—C62—C63	−0.4 (3)
N2—C7—C8—O1	178.20 (18)	O3—C61—C62—N9	−2.2 (3)
C8—O1—C9—C10	163.49 (17)	C66—C61—C62—N9	−178.1 (2)
Zn—O1—C9—C10	2.7 (2)	O4—N9—C62—C63	142.8 (3)
C16—N3—C10—N4	0.4 (2)	O5—N9—C62—C63	−37.7 (3)
Zn—N3—C10—N4	168.07 (13)	O4—N9—C62—C61	−39.2 (3)
C16—N3—C10—C9	−179.99 (19)	O5—N9—C62—C61	140.2 (2)
Zn—N3—C10—C9	−12.4 (3)	C61—C62—C63—C64	2.6 (4)
C11—N4—C10—N3	0.2 (2)	N9—C62—C63—C64	−179.6 (2)
C24—N4—C10—N3	−174.32 (18)	C62—C63—C64—C65	−4.3 (3)
C11—N4—C10—C9	−179.35 (19)	C62—C63—C64—N10	173.9 (2)
C24—N4—C10—C9	6.1 (3)	O7—N10—C64—C63	−156.3 (2)
O1—C9—C10—N3	5.4 (3)	O6—N10—C64—C63	22.0 (3)
O1—C9—C10—N4	−175.12 (19)	O7—N10—C64—C65	21.9 (3)
C10—N4—C11—C12	177.9 (2)	O6—N10—C64—C65	−159.8 (2)
C24—N4—C11—C12	−7.4 (4)	C63—C64—C65—C66	3.8 (3)
C10—N4—C11—C16	−0.8 (2)	N10—C64—C65—C66	−174.4 (2)
C24—N4—C11—C16	173.88 (19)	C64—C65—C66—C61	−1.5 (3)
N4—C11—C12—C13	−179.2 (2)	C64—C65—C66—N11	−176.59 (19)
C16—C11—C12—C13	−0.7 (4)	O3—C61—C66—C65	−176.0 (2)
C11—C12—C13—C14	−0.7 (4)	C62—C61—C66—C65	−0.2 (3)
C12—C13—C14—C15	1.3 (4)	O3—C61—C66—N11	−1.0 (3)
C13—C14—C15—C16	−0.5 (4)	C62—C61—C66—N11	174.88 (19)
C14—C15—C16—N3	178.3 (2)	O8—N11—C66—C65	35.6 (3)
C14—C15—C16—C11	−0.9 (3)	O9—N11—C66—C65	−144.5 (2)
C10—N3—C16—C15	179.8 (2)	O8—N11—C66—C61	−139.9 (2)
Zn—N3—C16—C15	13.5 (3)	O9—N11—C66—C61	40.0 (3)
C10—N3—C16—C11	−0.9 (2)	O12—N12—C68—C69	35.4 (3)
Zn—N3—C16—C11	−167.23 (14)	O11—N12—C68—C69	−144.6 (2)
C12—C11—C16—C15	1.5 (3)	O12—N12—C68—C67	−143.5 (2)
N4—C11—C16—C15	−179.6 (2)	O11—N12—C68—C67	36.5 (3)
C12—C11—C16—N3	−177.8 (2)	O10—C67—C68—C69	−177.1 (2)
N4—C11—C16—N3	1.1 (2)	C72—C67—C68—C69	2.3 (3)
C7—N2—C17—C18	107.5 (2)	O10—C67—C68—N12	1.7 (4)
C6—N2—C17—C18	−74.6 (3)	C72—C67—C68—N12	−178.9 (2)
N2—C17—C18—C19	−49.2 (3)	N12—C68—C69—C70	177.2 (2)
N2—C17—C18—C23	134.1 (2)	C67—C68—C69—C70	−4.0 (4)
C23—C18—C19—C20	0.0 (3)	C68—C69—C70—C71	2.4 (3)
C17—C18—C19—C20	−176.7 (2)	C68—C69—C70—N13	−174.4 (2)
C18—C19—C20—C21	−1.4 (4)	O14—N13—C70—C71	−3.1 (3)
C19—C20—C21—C22	1.5 (4)	O13—N13—C70—C71	177.6 (2)
C20—C21—C22—C23	−0.3 (4)	O14—N13—C70—C69	173.8 (2)
C19—C18—C23—C22	1.2 (3)	O13—N13—C70—C69	−5.6 (3)
C17—C18—C23—C22	178.0 (2)	C69—C70—C71—C72	0.7 (3)
C21—C22—C23—C18	−1.1 (4)	N13—C70—C71—C72	177.5 (2)
C10—N4—C24—C25	103.3 (2)	C70—C71—C72—N14	179.3 (2)
C11—N4—C24—C25	−70.3 (3)	C70—C71—C72—C67	−2.4 (3)
N4—C24—C25—C30	115.6 (2)	O16—N14—C72—C71	−164.2 (2)
N4—C24—C25—C26	−66.7 (3)	O15—N14—C72—C71	15.7 (3)
C30—C25—C26—C27	0.6 (3)	O16—N14—C72—C67	17.4 (3)
C24—C25—C26—C27	−177.2 (2)	O15—N14—C72—C67	−162.6 (2)
C25—C26—C27—C28	0.3 (3)	O10—C67—C72—C71	−179.6 (2)
C26—C27—C28—C29	−0.6 (4)	C68—C67—C72—C71	1.0 (3)
C27—C28—C29—C30	0.0 (4)	O10—C67—C72—N14	−1.4 (4)
C26—C25—C30—C29	−1.2 (3)	C68—C67—C72—N14	179.22 (19)
C24—C25—C30—C29	176.5 (2)	C75—N15—C73—O17	−178.0 (2)
C28—C29—C30—C25	0.9 (4)	C74—N15—C73—O17	1.1 (4)
C37—N5—C31—C32	179.4 (2)	C77—N16—C76—O18	−1.7 (5)
Zn—N5—C31—C32	−3.0 (3)	C78—N16—C76—O18	−178.5 (3)
